# Topic modeling and social network analysis approach to explore diabetes discourse on Twitter in India

**DOI:** 10.3389/frai.2024.1329185

**Published:** 2024-02-12

**Authors:** Thilagavathi Ramamoorthy, Vaitheeswaran Kulothungan, Bagavandas Mappillairaju

**Affiliations:** ^1^School of Public Health, SRM Institute of Science and Technology, Kattankulathur, Tamil Nadu, India; ^2^ICMR-National Centre for Disease Informatics and Research, Bengaluru, India; ^3^SRM Institute of Science and Technology, Kattankulathur, Tamil Nadu, India; ^4^Centre for Statistics, SRM Institute of Science and Technology, Kattankulathur, Tamil Nadu, India

**Keywords:** diabetes, social media, Twitter, India, content analysis, network analysis, machine learning, topic modeling

## Abstract

**Introduction:**

The utilization of social media presents a promising avenue for the prevention and management of diabetes. To effectively cater to the diabetes-related knowledge, support, and intervention needs of the community, it is imperative to attain a deeper understanding of the extent and content of discussions pertaining to this health issue. This study aims to assess and compare various topic modeling techniques to determine the most effective model for identifying the core themes in diabetes-related tweets, the sources responsible for disseminating this information, the reach of these themes, and the influential individuals within the Twitter community in India.

**Methods:**

Twitter messages from India, dated between 7 November 2022 and 28 February 2023, were collected using the Twitter API. The unsupervised machine learning topic models, namely, Latent Dirichlet Allocation (LDA), non-negative matrix factorization (NMF), BERTopic, and Top2Vec, were compared, and the best-performing model was used to identify common diabetes-related topics. Influential users were identified through social network analysis.

**Results:**

The NMF model outperformed the LDA model, whereas BERTopic performed better than Top2Vec. Diabetes-related conversations revolved around eight topics, namely, promotion, management, drug and personal story, consequences, risk factors and research, raising awareness and providing support, diet, and opinion and lifestyle changes. The influential nodes identified were mainly health professionals and healthcare organizations.

**Discussion:**

The study identified important topics of discussion along with health professionals and healthcare organizations involved in sharing diabetes-related information with the public. Collaborations among influential healthcare organizations, health professionals, and the government can foster awareness and prevent noncommunicable diseases.

## 1 Introduction

Diabetes was ranked as the eighth leading cause of mortality and morbidity globally in 2019 (GBD 2019 Diseases and Injuries Collaborators, 2020). There were an estimated 529 million individuals of all ages across the globe in 2021, leading to one in 17 individuals with diabetes, posing a severe burden to healthcare systems. South Asia constitutes around 20% of the global burden of diabetes in 2021 (Afroz et al., 2018; GBD 2021 Diabetes Collaborators, 2023). India is currently undergoing an epidemiological transition due to an upsurge in the prevalence of noncommunicable diseases, contributing 76.5% to the diabetes burden in the South Asia region in 2021 (The World Bank, 2013; Siegel et al., 2014). Of note, 1 in 9 adults and 1 in 7 adults aged 20 years and older were found to be diabetic and prediabetic, respectively, in 2021, with the prevalence higher among urban and male populations compared to rural and female adults (Anjana et al., 2023). Prevention, early diagnosis, and treatment are the key components for the reduction of the burden of diabetes. Efficient dissemination of health information on the prevention and control of diabetes leads to improved behavior and lifestyle changes, positively impacting both behavioral and metabolic risk factors (White et al., 2014; Haghravan et al., 2021). India is striving to achieve the sustainable development goal target of one-third reduction (Kulothungan et al., 2023). The initial step in diabetes prevention and control is to enhance the knowledge, attitudes, and perceptions of the general public, patients, and healthcare providers regarding awareness, treatment, and adherence (Tripathy et al., 2019).

In the contemporary landscape, social media has become an integral facet of people's daily existence, reshaping the way individuals interact, disseminate information, and connect with one another. A key catalyst for the expansion of social media is the widespread availability of Internet services and the proliferation of smartphones. As of April 2023, the global count of Internet users reached 5.18 billion, encompassing 64.6% of the world's population. Of them, a substantial number of individuals, 4.8 billion or 59.9% of the global population, actively engage with social media platforms (Petrosyan, 2023). Social media platforms, such as Facebook, Twitter, and Instagram, serve as a valuable forum for patients, healthcare professionals, community members, policymakers, and researchers to participate in conversations related to health matters. These platforms foster a dynamic and cost-effective environment for robust health communication (Moorhead et al., 2013; Smailhodzic et al., 2016).

Previous studies have shown that health departments, patients, healthcare professionals, and patient groups use social media for health communication regarding diabetes management and control, but this usage is still in its early stages (Park et al., 2015; Liu et al., 2016; Alanzi, 2018; Gavrila et al., 2019; Da Moura Semedo et al., 2023). Qualitative investigations into diabetes-related groups on Facebook and online blogs revealed that they positively impact patients and caregivers by enhancing their knowledge, allowing them to share personal experiences, and seeking technical and social support (Greene et al., 2011; Staite et al., 2018; Stellefson et al., 2019). In the context of Twitter, scholars such as Karami et al. (2018) and Shaw and Karami (2017) have utilized computational methodologies, including techniques like topic modeling and linguistic analysis, to gain insights into the sentiments and opinions expressed by users concerning diabetes, dietary choices, and obesity in the Twitter discourse. Similarly, Beguerisse-Díaz et al. (2017) analyzed the content of Twitter messages and influential users using a mixed-methods approach, including thematic analysis, network analysis, and topic detection. Their study found that diabetes-related interactions on Twitter revolved around information, news, social interaction, and commercial content. Furthermore, studies have incorporated lexicon-based sentiment analysis techniques to evaluate the emotional tone conveyed by individuals in their discussions about diabetes on Twitter. Gabarron et al. (2019), for instance, performed sentiment analysis on diabetes-related tweets and discovered that tweets about type II diabetes, particularly those without emojis, tended to be negative, while those about type I diabetes were more positive. Further investigations into Twitter conversations about type I diabetes revealed that a significant number of tweets came from individuals affected by diabetes, non-governmental organizations, and media sources (Gabarron and Makhlysheva, 2015). Liu et al. (2016) also observed that diabetes-related participation on Twitter was increasing and studied the temporal patterns in diabetes tweets. They found that there was high seasonality, with a surge in tweets during November, coinciding with World Diabetes Day. However, geolocation information was relatively scarce in the tweets. Lenzi and Iazzetta (2023) mapped the representation of obesity and diabetes on Twitter for Italy and found that they are correlated with each other, indicating the intertopic nature of health. Information published in the form of videos has been analyzed for its effectiveness in increasing awareness about various aspects of diabetes (Erten, 2022; AlBloushi and Abouammoh, 2023; Rana and Arora, 2023).

For effective and accurate dissemination of diabetes information on social media to a broader audience, it is essential to analyze and comprehend the nature, content, and structure of messages shared by the public and healthcare professionals. This understanding will help identify and address misinformation while developing targeted strategies to promote reliable and evidence-based health information. As health information-seeking behavior is different across the globe, it is essential to understand social media usage for health-related information, its content, types of users, sentiments, and social networks for developing strategic interventions for the community (Raamkumar et al., 2016). Despite the growing importance of social media in health communication, research on how it is utilized for diabetes-related discourse in India is notably scarce (Diviya Prabha and Rathipriya, 2022; Karmegam, 2022).

Many natural language processing, machine learning, and topic modeling techniques have been developed to uncover information from unstructured data (Murshed et al., 2023). Most of these techniques require programming skills, but improvements in the user-friendly coding software have enabled their widespread use (Yu and Egger, 2021). However, proper tuning of the model parameters is essential, for which there is a lack of clear guidance. Various topic models exist, including conventional ones like Latent Dirichlet Allocation (LDA) and non-negative matrix factorization (NMF), as well as emerging models like Top2Vec and BERTopic (Chen et al., 2023). In medical and social science, traditional and conventional algorithms are increasingly used over newer methods due to a lack of knowledge about these techniques or their implementation (Egger and Yu, 2021).

With this background, this study analyzed the diabetes-related discussions on Twitter in India by extracting the topics of conversation and identifying the key influencers using various content analysis techniques as well as network analysis. The study aimed (1) to examine and compare the performance of conventional methods such as LDA and NMF, (2) to evaluate and compare the performance of emerging methods such as Top2Vec and BERTopic, (3) to identify the prevalent topics within Twitter conversations related to diabetes in India, (4) to measure the extent of outreach and user engagement in diabetes-related tweets, (5) to visualize and characterize the diabetes discussion network, and (6) to identify the influencers behind the diabetes-related discussions on Twitter in India. This research sheds light on analyzing unstructured text data from social media using machine learning methods. The findings of this research have the potential to significantly contribute to our understanding of social media's role in diabetes management and health communication in India, informing targeted interventions and strategies for public health improvement. By unraveling the nature, content, and structure of diabetes-related messages on social media, this study seeks to provide insights that can guide the development of evidence-based health information. The implications of these findings extend to the formulation of effective public health strategies for diabetes management and awareness in the Indian context.

## 2 Methodology

### 2.1 Data collection

**Figure 1:**
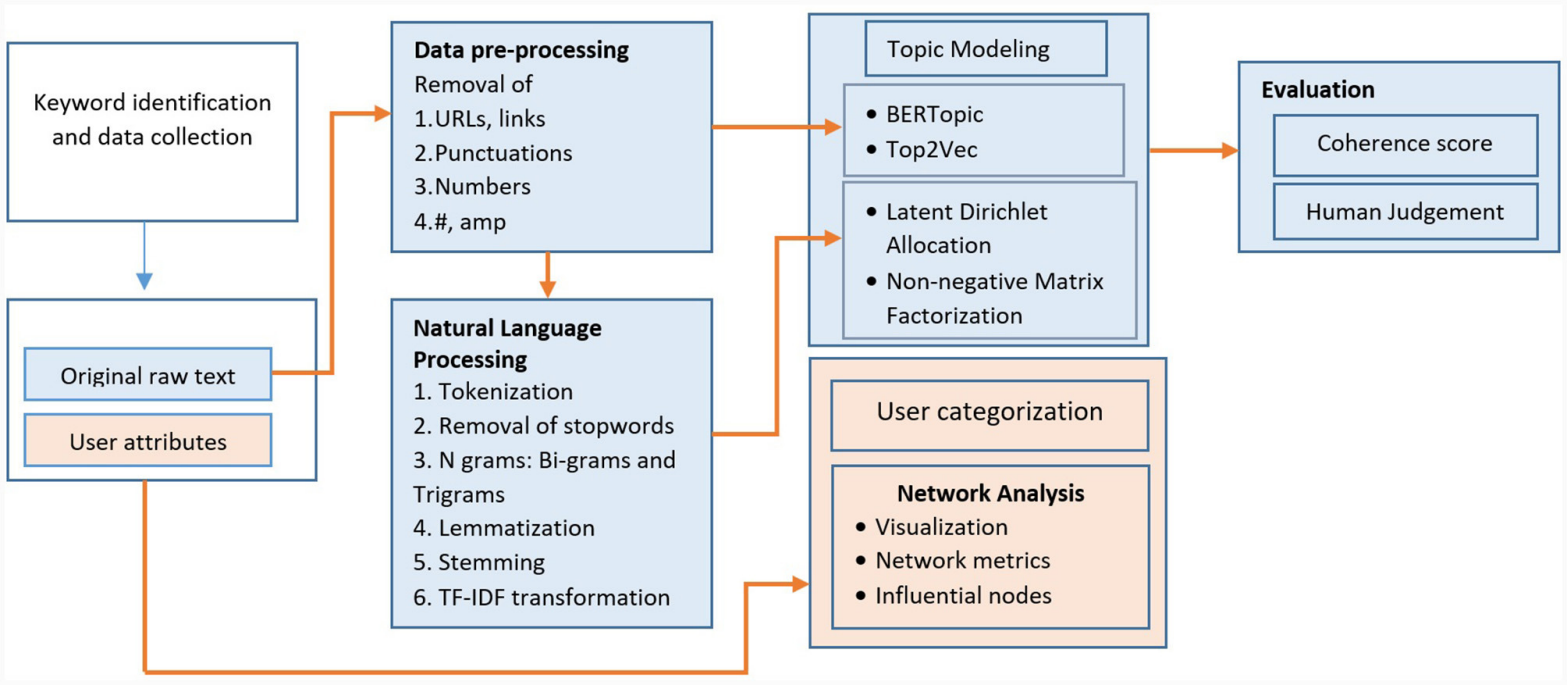
Methodological framework of Twitter Diabetes data analysis.

Twitter is a widely used social media platform for sharing small pieces of information on a global scale, and it serves as a valuable source of data for conducting content and network analyses. The keywords associated with diabetes were chosen from the Symplur Healthcare Hashtag Project, which curates a compilation of hashtags relevant to various diseases and health conditions (The Healthcare Hashtag Project, 2023). The keywords include “diabetes”, “diabetesmellitus”, “T1D”, “diabetic”, “diabeticawareness”, “diabetesmanagement”, “diabetesfood”, “diabeteslifestyle”, and “India”. The tweets posted between 7 November 2022 and 28 February 2023 were obtained using Twitter's REST Application Program Interface through the R package “rtweet”. Publicly available messages that contain at least one of the keywords related to diabetes along with user information were retrieved (Twitter Developer, 2023). Tweets were gathered at weekly intervals, with varying combinations of keywords and hashtags used to refine the data collection process. The collected tweets were consolidated, and duplicates were detected by examining the text and the user's screen name. Tweets originating from locations outside of India and tweets not in the English language were eliminated from the dataset. The analysis framework is illustrated in Figure 1.

### 2.2 Data pre-processing for text analysis

Before analyzing the retrieved tweets, several pre-processing steps were carried out, which included (1) eliminating non-English words; (2) converting all words to lowercase for stemming purposes; (3) removing stop words; (4) removing specific words such as “amp”, “https”, “that”, and “will”; (5) removing Uniform Resource Locators and links; and (6) stemming inflected words into their roots and completing the stemming process to obtain complete words (tokens) for visualization purposes. Additionally, duplicate tweets were removed at this stage. To perform these pre-processing steps, various Python libraries were employed, including nltk for natural language processing; seaborn and matplotlib for data visualization; wordcloud for word visualization; sklearn for data transformation, testing, and model fitting; re for regular expressions; and os for identifying file locations.

### 2.3 Implementation of different topic models

Topic models are statistical models designed to identify clusters of words that effectively encapsulate the information contained within a document, facilitating the natural categorization and grouping of documents (Probabilistic Topic Models, 2021).

#### 2.3.1 Latent Dirichlet Allocation

Latent Dirichlet Allocation (LDA) stands as a well-recognized unsupervised machine learning approach for topic modeling, unveiling latent themes within text data (Blei et al., 2003). This method has found extensive application in unveiling concealed themes related to various diseases and health conditions (Ghosh and Guha, 2013; Tapi Nzali et al., 2017; Cesare et al., 2020; Valdez et al., 2020; Zhou et al., 2020). When representing the tweet messages as M, the words within those tweets as W, and the predetermined number of topics as K, LDA yields two key probabilities: P(t|m), which signifies the probability of words within tweet messages (m) being assigned to topic (t), and P(w|t), representing the probability of topic (t) within the entire collection of tweet messages for the word (w). The hyperparameters α and β dictate the prior distributions for these probabilities.

For the implementation of LDA topic modeling, the Python libraries Spacy and Gensim were utilized. The tuning parameter λ was set within the range of 0.6 to 0.9, and LDA was executed with 3,000 Gibbs sampling iterations. The optimal number of topics for the LDA model was determined using the coherence score, which quantifies the similarity between different topics by computing the average cosine values between every pair of context vectors (Stevens et al., 2012). The exploration for the ideal number of topics commenced with a range from two to fifteen, incrementing one at a time. A higher coherence score is indicative of a better number of topics, and in this case, the optimal number was found to be 8 (as shown in Appendix Figure 1).

Within each identified topic, the top 10 words with the highest beta values were reported, and the topics were named based on these keywords. LDAvis was employed for visually representing the intertopic distance map (as displayed in Appendix Figure 2). Additionally, the t-distributed stochastic neighbor embedding (t-SNE) algorithm was used to visualize the clusters of documents, as presented in Appendix Figure 3.

#### 2.3.2 Non-negative matrix factorization

In contrast to LDA, Non-negative matrix factorization (NMF) is a non-probabilistic algorithm that employs matrix factorization and falls into the category of linear algebraic methods (Egger, 2022b). NMF operates on Term Frequency-Inverse Document Frequency (TF-IDF)-transformed data by decomposing a matrix into two lower-ranked matrices (Obadimu et al., 2019). Term Frequency-Inverse Document Frequency (TF-IDF) measures the importance of words in a document collection. NMF decomposes its input, which is typically a term-document matrix (denoted as A), into the product of two separate matrices: a terms-topics matrix (represented as W) and a topics-documents matrix (expressed as H). This factorization process is a fundamental step in NMF, enabling the extraction of underlying patterns and relationships within the original data. W contains basis vectors, and H contains corresponding weights, with both matrices of non-negative values being iteratively adjusted (Chen et al., 2018).

Both LDA and NMF require preprocessed data, and as part of the process, natural language processing was performed. The coherence score indicated that the number of topics in NMF was 10 (Appendix Figure 1). Following topic modeling, a manual evaluation of the contents of 20% of the tweets in each identified topic was performed to understand the perception of the population, the validity of the topic, and the grouping of the tweets in the topic. After analyzing the most commonly used words and sample tweets, and through discussion, each theme was given a concise title.

#### 2.3.3 Top2Vec

Top2Vec (Angelov, 2020) is a relatively recent algorithm harnessing word embeddings. This approach involves converting text data into vectors, enabling the identification of semantically related words, sentences, or documents in close spatial proximity (Egger, 2022a). For instance, words like “cancer” and “tumor” should exhibit closer proximity than words like “cancer” and “diet”. In this research, pre-trained embedding models, namely, All-MiniLM-L6-v2, Doc2Vec, and Universal Sentence Encoder, were compared based on the coherence score, and word and document embeddings were generated. Since word vectors that are closely aligned with document vectors tend to provide the most accurate representation of a document's underlying topic, the number of documents that can be grouped together indicates the number of distinct topics (Hendry et al., 2021).

To address the inherent sparsity often encountered in vector spaces, a dimension reduction step was executed prior to density clustering. This entailed employing uniform manifold approximation and projection (UMAP) to reduce the dimensionality to a level conducive to the application of hierarchical density-based spatial clustering of applications with noise (HDBSCAN). The objective was to identify densely populated regions within the documents (Angelov, 2020). Following this, the centroid of the document vectors in the original high-dimensional space was computed for each of these dense regions, effectively representing the topic vector.

It is important to note that words appearing in multiple documents were identified as noise by HDBSCAN since they cannot be assigned to a single document. As a result, Top2Vec eliminates the need for preprocessing steps like removing stopwords or performing stemming and lemmatization (Ma et al., 2021; Thielmann et al., 2021). Therefore, Top2Vec autonomously offers insights into the number of topics, the sizes of these topics, and the words that define each individual topic.

#### 2.3.4 BERTopic

BERTopic is a topic modeling technique that harnesses the capabilities of BERT (bidirectional encoder representations from transformers), a state-of-the-art transformer-based natural language processing model (Grootendorst, 2020). Similar to Top2Vec, BERTopic also uses UMAP for dimension reduction and HDBSCAN for document clustering. The word embeddings was generated based on the following models, namely, LaBSE, paraphrase-MiniLM-L3-v2, all-MiniLM-L6-v2, all-MiniLM-L12-v2, all-mpnet-base-v2, universal sentence encoder, and random-nnlm-endim50. BERTopic, like Top2Vec, diverges from LDA by providing a form of continuous topic modeling rather than discrete (Alcoforado et al., 2022). This inherent stochasticity in the model results in varying outcomes upon repeated modeling runs. Once the model is computed, researchers can extract the most significant topics. It is worth noting that Topic 0, marked with a count of −1, consistently represents outliers and should not be subjected to further analysis. Additionally, researchers have the option to search for specific keywords and receive the most relevant topics based on their similarity scores. They can also delve into individual topics by examining their associated keywords.

To ease the exploration of the potentially vast range of topics, BERTopic provides an interactive intertopic distance map, which allows for the examination of each topic individually (Grootendorst, 2020). As illustrated in Figure 3, following the initial overview of the topics, researchers have the option to conduct automated topic reduction to further refine the set of topics.

### 2.4 Source and reachability evaluation

The origin of each tweet was scrutinized and manually grouped into seven categories: individual, individual health, healthcare organization, hospital, media, non-health organization, and others. The accessibility of these tweets was assessed through two metrics: “favorite tweets,” which represents the number of likes a tweet received, and “re-tweet count,” which signifies the number of times a tweet was shared. These metrics serve as indicators of the tweets' popularity and convey the level of interaction among users, with larger numbers indicating a broader reach.

To analyze reachability in a topic-specific and user-type context, we calculated a ratio. This ratio is determined by dividing either the re-tweet count or favorite count by the total number of tweets within the corresponding topic or user type. This calculation provides insights into how effectively tweets within specific topics or from specific user types are being disseminated and engaged with by the audience.

### 2.5 Network analysis

A social network analysis was performed to identify the influential nodes that are important in spreading information related to diabetes. The Twitter user information and the metadata were used to develop a user-retweet network with the users as the nodes and the tweet communications between the users as the edges. This analysis considered retweets for the creation of a directed network. In a retweet connection between A and B, when A retweets a tweet from B, a connection is formed from A to B. Network centrality measures such as indegree, outdegree betweenness, closeness, and eigenvector centrality were calculated (Hage and Harary, 1995). Degree centrality refers to the number of connections of a node, and those nodes with high degree centrality are well-connected and often considered influential because they can reach a large portion of the network directly. Indegree measures how many times a user's tweets have been retweeted by other users. Users with a high indegree are considered influential, as their content is being shared widely across the network. Outdegree, in the context of a retweet network, represents the number of retweets a user has made or the number of times a user has retweeted other users' content. It measures the extent to which a user actively shares or propagates information by retweeting other users' tweets. A high outdegree suggests that a user actively contributes to the dissemination of content. In some cases, the concept of weighted degree might be used to account for the strength or significance of connections in the retweet network. In a weighted network, each connection (or edge) between users is assigned a weight, which could represent the number of times one user has retweeted another user's content. Therefore, the weighted degree would take into account not just the number of connections (retweets) but also the strength or frequency of those connections.

Betweenness centrality quantifies the number of times a node lies on the shortest path between other pairs of nodes. Nodes with high betweenness centrality can control or influence the flow of information between different parts of the network. Closeness centrality quantifies how rapidly a node can establish connections with all other nodes within a network. Nodes with high closeness centrality are considered influential because they can efficiently spread information or influence across the network. Eigenvector centrality considers not only a node's connections but also the centrality of its neighbors. Nodes connected to other influential nodes will have higher eigenvector centrality. It is similar to the idea that your influence increases if you are connected to influential people. PageRank measures the importance of a node based on the links to it and the importance of the nodes that link to it. Nodes with a high PageRank are considered influential because they are connected to other influential nodes. These measures were used to identify the influential users in the diabetes network. The Gephi software (version 0.10.1) was used for this analysis and visualization. The size of the node denotes the average weighted degree, with degrees ranging from 2 to 2,770, and laid out using the Fruchterman-Reingold layout and the Force Atlas 2 algorithm. The network characteristics and top influential users were presented.

### 2.6 Ethical approval

The study received ethical approval from the institutional ethics review committee at SRM Medical College Hospital and Research Center, SRM Institute of Science and Technology.

## 3 Results

There were a total of 59,159 diabetes tweets generated during the 15-week study period. The distribution of tweets across the weeks was presented in Appendix Figure 4. Out of the total tweets, 33.0% of them were unique tweets (Table 1).

**Table 1:** Key diabetes tweet descriptors in India for the period November 2022 to February 2023.

**Data descriptor**	**Total count**	**%**
Tweets generated	59,159	
Tweets with unique messages	19,533	33.0
Tweets with mentions	30,508	51.6
Tweets that were retweets	16,337	27.6
Tweets with links	19,972	33.8
Tweets with media (photo, video)	15,708	26.6
Tweets that were replies	10,165	17.2

### 3.1 Content analysis

#### 3.1.1 Comparison of LDA and NMF

The first step in LDA or NMF topic modeling is the identification of the optimal number of topics. The coherence score was used as the metric to identify the optimal number of topics. Appendix Figure 1 shows that the highest coherence score was noted for the LDA model for topic 8. Also, the manual evaluation revealed the topics identified in both models as presented in Table 2. Topics in the LDA model are more diverse, with the same content being discussed in multiple topics. Hence, this study identified that NMF outperforms LDA in identifying the latent topics related to diabetes on Twitter in an Indian context.

**Table 2:** Comparison of topics between LDA and NMF.

**Topic no**	**NMF**	**Topic no**	**LDA**
Topic 1	Promotion	Topic 1	Promotion
Topic 2	Management, Drug and personal story	Topic 3	Personal story, consequences
Topic 3	Consequences	Topic 3 and 5	Consequences
Topic 4	Risk factors and research	Topic 7	Risk factors
Topic 5	Raising awareness, and providing support	Topic 6	Awareness
Topic 6	Diet	Topic 2, 4 and 8,10	Diet, Drug and lifestyle
Topic 7	Opinion	Topic 3	Diet, opinion, personal story
Topic 8	Lifestyle changes	Topic 9	Lifestyle, treatment, diet, consequences

**Figure 2:**
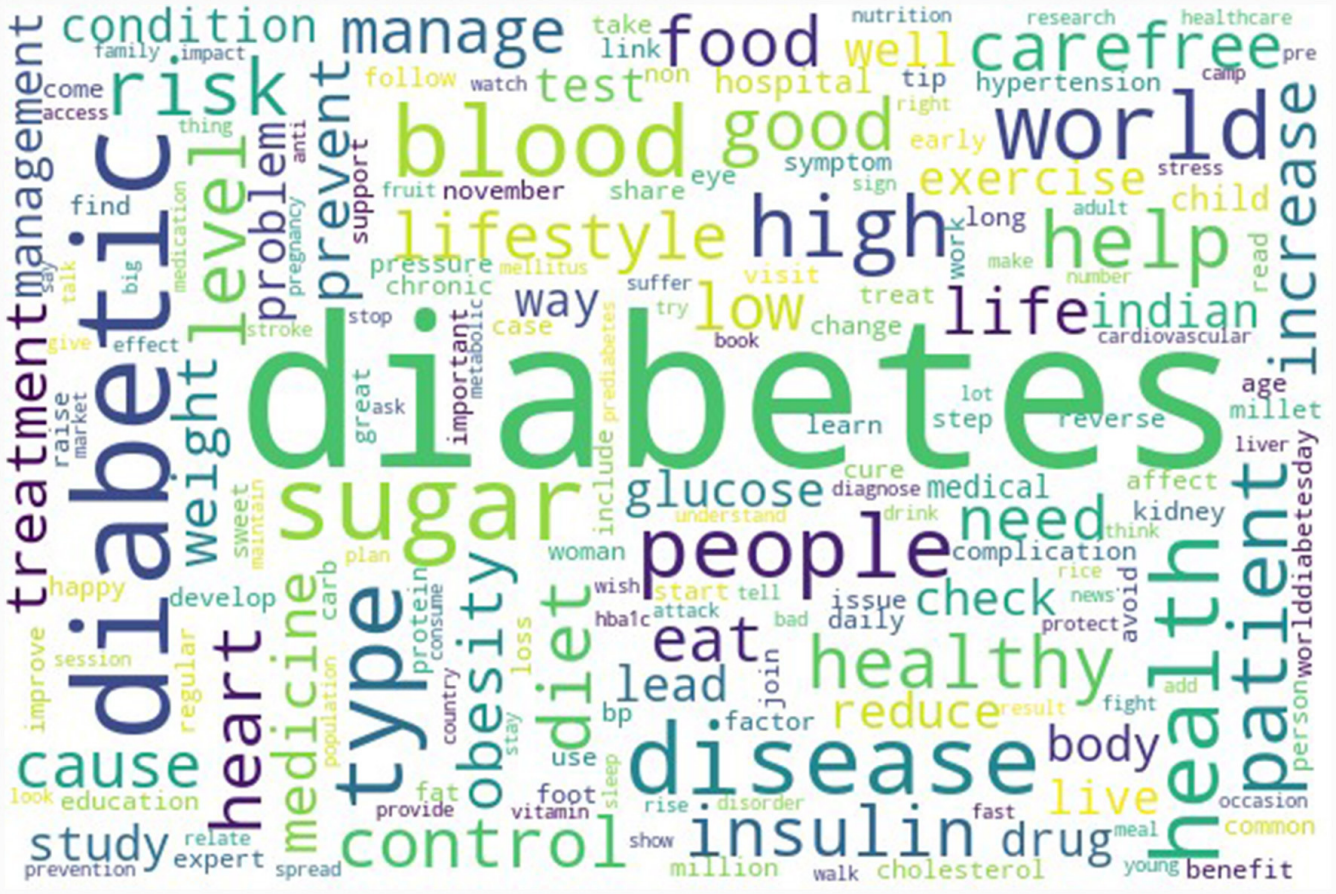
Wordcloud of top 300 word tokens in diabetes related tweets in India during November 2022 to February 2023.

**Figure 3:**
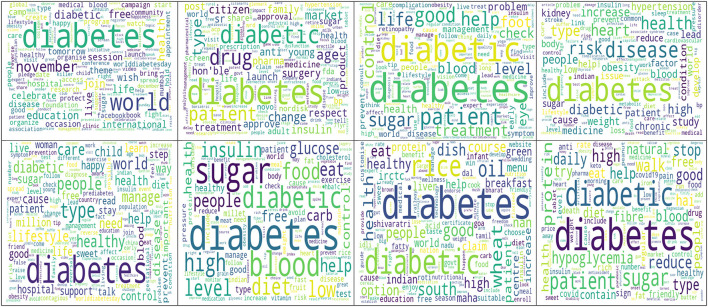
Topicwise word cloud of top 100 words in diabetes related tweets in India during November 2022 to February 2023. Top left to right: Topic 1 to Topic 4; Bottom left to right: Topic 5 to Topic 8.

Figure 2 presents the top 300 words present in the entire diabetes tweet corpus. Table 3 presents the topics along with the distribution of topics by types of Twitter users. Every one out of four diabetes-related tweets was about risk factors and research-related tweets. Information about risk factors such as smoking, obesity, and lack of physical activity, along with comorbidities such as hypertension, was shared on this topic. The study findings and the articles related to diabetes were also widely disseminated through this platform. The other prominent topics identified were raising awareness and providing support (22.5%), diet (18.6), consequences (14.9%), and promotion (10.4%). The topic-wise top words are presented in Figure 3. Four out of 10 diabetes tweets originated from individuals, followed by individuals from health background (including doctors). Around 30% of the promotion-related tweets were from healthcare organizations and hospitals (Appendix Figure 5). The beta values of top 50 words for each topic are reported in Appendix Table 1.

**Table 3:** Identified topics, example tweets, and distribution of tweets by type of user for diabetes.

**Topic**	**Example tweets**	**n (%)**	**Type of user (source) n (%)**						
			**Individual**	**Individual-Health**	**Healthcare organization**	**Hospital**	**Media**	**Non-health organization**	**Others**
Promotion	Grab our #diabetic #porridge (Delight Medley PRO) made with quality and passion that does not trigger your carb appetite and is preferably made with handpicked cereals, luscious legumes, in-house dehydrated fruits, and more. #porridgemix	2,024 (10.4)	529 (26.1)	462 (22.8)	494 (24.4)	137 (6.8)	207 (10.2)	195 (9.6)	(0)
Management, Drug and personal story	World Diabetes Day 2022. DiaCinn, a promising supplement for pre-diabetic and diabetic management.Learn more at xxx. #nutrispice #Worlds2022 #diabetes #DiabetesAwarenessMonth #diabetesday #DiabetesAwareness #DiabetesCare #healthcare #health #TrendingN	815 (4.17)	353 (43.3)	151 (18.5)	99 (12.1)	10 (1.2)	160 (19.6)	41 (5.0)	1 (0.1)
	Acupressure is an alternative healing method to treat several diseases such as cure diabetes naturally without side effects. Discover more here on this #DiabetesAwarenessMonth								
	Triphala is a wonderful Ayurvedic remedy. It is made of Amla, Harad and Baheda. The most significant benefits of Triphala include improving digestion, reducing signs of aging, detoxifying the body, aiding weight loss, strengthening the immune system and managing diabetes.								
	Joined ArtofLiving, daily doing Surya Namaskar, Yogas, twice a day and #sudarshankriya. Reduced wt from 130 to 84, reversed the diabetes. Best one everyone should join								
Consequences	Diabetic Retinopathy is sometimes IRREVERSIBLE, but yearly eye check-ups can reduce the risk of BLINDNESS by 95%. Get your eyes checked now. Free RBS test on the occassion of world diabetes day	2,919 (14.9)	1,169 (40.0)	564 (19.3)	436 (14.9)	176 (6.0)	384 (13.2)	188 (6.4)	2 (0.1)
Risk factors and research	Are you aware that smoking is a risk factor for type 2 diabetes? It is more likely to develop if you smoke. In fact, smoking can make it more difficult to maintain blood sugar control if you are already diabetic.	4,678 (23.9)	1,492 (31.9)	1,112 (23.8)	719 (15.4)	201 (4.3)	837 (17.9)	314 (6.7)	3 (0.1)
	WHAT ARE RISK FACTORS FOR DIABETES? - Zee News #diabetes #vingscommunity #news								
Raising awareness, and providing support	Hypoglycemia is more dangerous compared to Hyperglycemia. Hypoglycemia will triggers the Seizures and Hypoglycemia unawareness.	4,401 (22.5)	1,828 (41.5)	818 (18.6)	723 (16.4)	228 (5.2)	497 (11.3)	290 (6.6)	17 (0.4)
	hi XYZ. Is sweet corn is good for diabetic. Actually in the evening time i am eating pumpkin seeds along with dry dates. I want to change my evening menu.								
	Hba1c of 7% in regular check ups of every 3 months and fbs, ppbs are normal should we consider it as diabetes, no h/o diabetes, hypertension.								
Diet	Want to maintain bloodsugar level? Here are 7 food items to avoid in Type 2 diabetes.	3,626 (18.6)	1,372 (37.8)	799 (22)	587 (16.2)	98 (2.7)	529 (14.6)	223 (6.2)	18 (0.5)
Opinion	Yes Dr. ABC, I fully agree with you. Educated diabetic patients start on alternative medicines and extol their virtue. The same patients after a couple of months, switch over to stronger allopathic medicines or insulin! I've seen it.	314 (1.61)	156 (49.7)	63 (20.1)	28 (8.9)	2 (0.6)	50 (15.9)	14 (4.5)	1 (0.3)
Lifestyle changes	How to control Diabetes without #Medicine #Diabetes is a wise spread #disease nowadays. But a little modification in lifestyle can even stop this fatal disease from growing. Know-how #health #life #DiabetesAwarenessMonth #DiabetesAwareness #HariOm	756 (3.87)	339 (44.8)	144 (19)	72 (9.5)	10 (1.3)	122 (16.1)	47 (6.2)	22 (2.9)
Total		19,533 (100.0)	7,238 (37.1)	4,113 (21.1)	3,158 (16.2)	862 (4.4)	2,786 (14.3)	1,312 (6.7)	64 (0.3)

#### 3.1.2 User type and reachability

One out of three unique tweets was from individuals, followed by individuals who identified themselves by their health background and healthcare organizations (16.2%). Around one out of seven tweets were from media outlets. Reachability analysis by the type of user revealed that tweets by individuals from health background were likely to be shared or retweeted an average of 3.2 times (retweet ratio: 3.2) and liked by an average of 17 users (favorite ratio: 17.4). The tweets about lifestyle changes had the highest favorite ratio (10.0), whereas the risk factors and research-related tweets had the highest retweet ratio (3.2) (Table 4).

**Table 4:** Retweet and favorite ratio across the topics identified in diabetes tweets in India.

**Topic/Type of user**	**Number of tweets**	**Retweet count**	**Favorite count**	**Retweet to total tweets ratio**	**Favorite to total tweets ratio**
**Topic**					
promotion	2,024	2,700	11,955	1.3	5.9
Management, Drug and personal story	815	1,791	6,133	2.2	7.5
consequences	2,919	8,978	21,171	3.1	7.3
risk factors and research	4,678	15,074	32,516	3.2	7.0
Raising awareness, and providing support	4,401	7,339	21,737	1.7	4.9
Diet	3,626	10,002	28,379	2.8	7.8
opinion	314	441	2,274	1.4	7.2
Lifestyle changes	756	2,156	7,558	2.9	10.0
**Type of user**					
Individual	7,238	22,890	30,838	3.2	4.3
Individual-Health	4,113	13,147	71,684	3.2	17.4
Healthcare organization	3,158	7,741	15,856	2.5	5.0
Hospital	862	417	1,214	0.5	1.4
Media	2,786	1,802	8,014	0.6	2.9
Non-health organization	1,312	2,482	4,108	1.9	3.1
Others	64	2	9	0.0	0.1

#### 3.1.3 Comparison of BERTopic and Top2Vec

Multiple embedding models in BERTopic and Top2Vec were examined, and the coherence scores indicated that the “all-MiniLM-L12-v2” has the highest coherence of 0.70. The transformer model and universal sentence encoder have a coherence score of 0.66, which is 4% less than the all-MiniLM-L12-v2. However, all the Top2Vec embedding models showed a much lower coherence score, as presented in Table 5.

**Table 5:** Coherence scores for different embedding models in BERTopic and Top2Vec.

	**Embedding Model**	**Coherence score**
BERTopic	LaBSE	0.61
	Paraphrase-MiniLM-L3-v2	0.60
	All-MiniLM-L6-v2	0.62
	All-MiniLM-L12-v2	0.70
	All-mpnet-base-v2	0.65
	Universal sentence encoder	0.66
	Random-nnlm-endim50	0.63
Top2Vec	All-MiniLM-L6-v2	0.28
	Doc2Vec	0.26
	Universal sentence encoder	0.26

BERTopic model with “all-MiniLM-L12-v2” as the embedder automatically generated 447 topics. The intertopic distance map is presented in Appendix Figure 6. Whereas the Top2Vec model with the “all-MiniLM-L6-v2” embedder generated 187 topics. The topics from the BERTopic model were grouped into 19 categories, such as diet (14.6%), promotion (14.1%), awareness (13.8%), risk factors (13.6%), and symptoms (10.8%). The 187 topics from the Top2Vec model were grouped into 19 categories. Notably, the majority of documents (41.1%) contributed to the “complication” topic, followed by diet (11.2%) and awareness (7.8%) (Table 6).

**Table 6:** Topic size and category names for the diabetes data using BERTopic and Top2Vec model for the diabetes tweets in India.

**Topic**	**BERtopic**			**Top2Vec**		
**No of documents**	**No of topics**	**% of documents**	**No of documents**	**No of topics**	**% of documents**
Diet	2,851	59	14.6	2,184	25	11.2
Promotion	2,753	54	14.1	1,201	10	6.1
Awareness	2,686	57	13.8	1,520	12	7.8
Risk factors	2,648	17	13.6	569	15	2.9
Symptoms	2,115	12	10.8	301	10	1.5
Complications	1,381	39	7.1	8,028	31	41.1
Treatment	990	20	5.1	611	12	3.1
Drug	952	26	4.9	549	17	2.8
Management	780	8	4.0	288	3	1.5
Statistics	740	10	3.8	770	6	3.9
Screening	385	5	2.0	164	15	0.8
Others	220	33	1.1	1,148	13	5.9
Research	193	25	1.0	517	3	2.6
Lifestyle	191	8	1.0	319	3	1.6
Diagnosis	175	6	0.9	203	2	1.0
Exercise	169	6	0.9	360	2	1.8
Personal story	145	15	0.7	214	2	1.1
Support	133	20	0.7	376	5	1.9
Opinion	26	21	0.1	211	1	1.1
Grand Total	19,533	441	100.0	19,533	187	100.0

**Figure 4:**
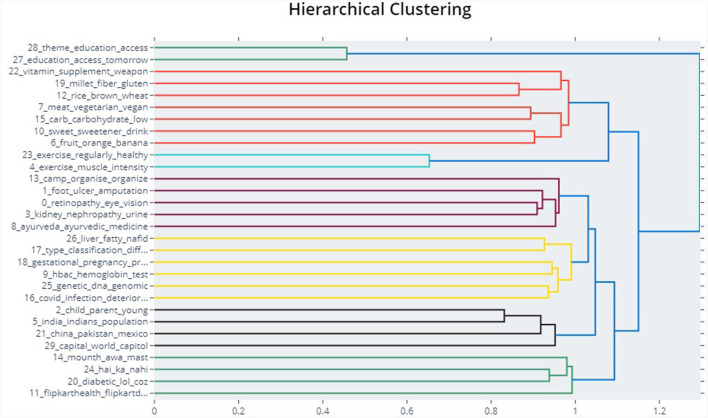
The hierarchical reduction of topics in BERTopic for diabetes related tweets in India.

Comparisons between the BERTopic and Top2Vec models were also performed using a specific search process for in-depth understanding and identification of the topics associated with the search keyword. For example, two terms were considered, namely, “foot” and “diet”. The BERTopic model revealed the topics relevant to the search term using cosine similarity. The top five topics associated with “foot” and “diet” were presented in Tables 7A, B. The BERTopic topics on “foot” appear to be more related to the complications of diabetes, research related to the complications, and generating awareness about lifestyle changes such as the diabetes-free run campaign. Similarly, a “Diet” search revealed topics closer to diet plan, vegetarian and non-vegetarian diet, types of diet, and awareness to avoid unhealthy diet. However, Top2Vec results for the same terms result in repeated topics such as unhealthy diet and unhealthy snack for diet and topics that include encouragement. Also, the dendrogram of the BERTopic shows the agglomeration of the individual topics (Figure 4).

**Table 7A:** Comparison between BERTopic and Top2Vec for Keyword “foot” in diabetes related tweets in India.

**BERTopic**		**Top2Vec**	
**Topic**	**Keyword**	**Topic**	**Keyword**
Foot ulcer	Foot, ulcer, amputation, wound, leg, footwear, limb, curafoot, healing, footcare	Amputation	Diabetologist, amputation, foot, leg, insulin, prediabetes, ulcer, neuropathy, hyperglycemia, wound
Foot numbness	Sensation, tingle, finger, numb, nerve, numbness, neuropathy, peripheral, disease, hand	Research	Article, explain, read, book, comment, discussion, understand, report, news, knowledge
Walk for cause	Walk, nation, came, tomorrow, easy, free, footpath, bicycle, lane, run	Obesity	Obese, diabetic, thirst, medication, diet, metabolic, nutritionist, cardiovascular, health, sugar
Free run	Finish, race, flipkartdiabetesfreerun, begin, step, wait, child, flipkarthealth, support, cure	Exercise for Fitness	Walk, exercise, obese, fitness, wellness, prediabetes, step, lifestyle, yoga, sedentary
Research	Read, article, asianjournalofmedicalscience, comment, medxlife, knowthedisease, click, explain, rebuttal, cart	Encouragement	Sweet, excellent, amazing, thanks, great, wonderfull, course, note, late, man, complete

**Table 7B:** Comparison between BERTopic and Top2Vec for Keyword “Diet” in diabetes related tweets in India.

**BERTopic**		**Top2Vec**	
**Topic**	**Keyword**	**Topic**	**Keyword**
Diet plan	Chart, diet, choosing, friendly, plan, struggle, calorie, dieting, preference, maintain	Unhealthy diet	Diet, obesity, unhealthy, diatary, nutrition, wellness, healthy, unhealthy, consume, eat
Vegetarian diet	Carbs, protein, dal, fiber, sabzi, roti, vegetarianism, avg, staple, food	Research	Read, article, explain, understand, book, discuss, comment, learn, answer, detail
Unhealthy diet	Eating, unhealthy, insecurity, esteem, habit, pattern, disrupt, refrain, consumption, food	Meal	Diabetic, diabetes, prediabetes, dietary, insulin, glycemic, meal, breakfast, calorie
Types of diet	Carbohydrate, mediterranean, keto, carbs, oggt, saturate, carnovore, dietary, protein, inherently	Unhealthy snack	Diabtetic, diabetes, snack, insulin, hyperglycemia, prediabetes, diet, glucose, nutrition, sugar
Nonvegetarian diet	Meat, vegetarian, vegan, diary, eater, map, animal, goat, egg, red	Vegetarian and non-vegetarian	Dietary, diabetes, nutrition, diet, meat, vegetarian, glycemic, wellness, wellness, food

### 3.2 Network analysis

**Figure 5:**
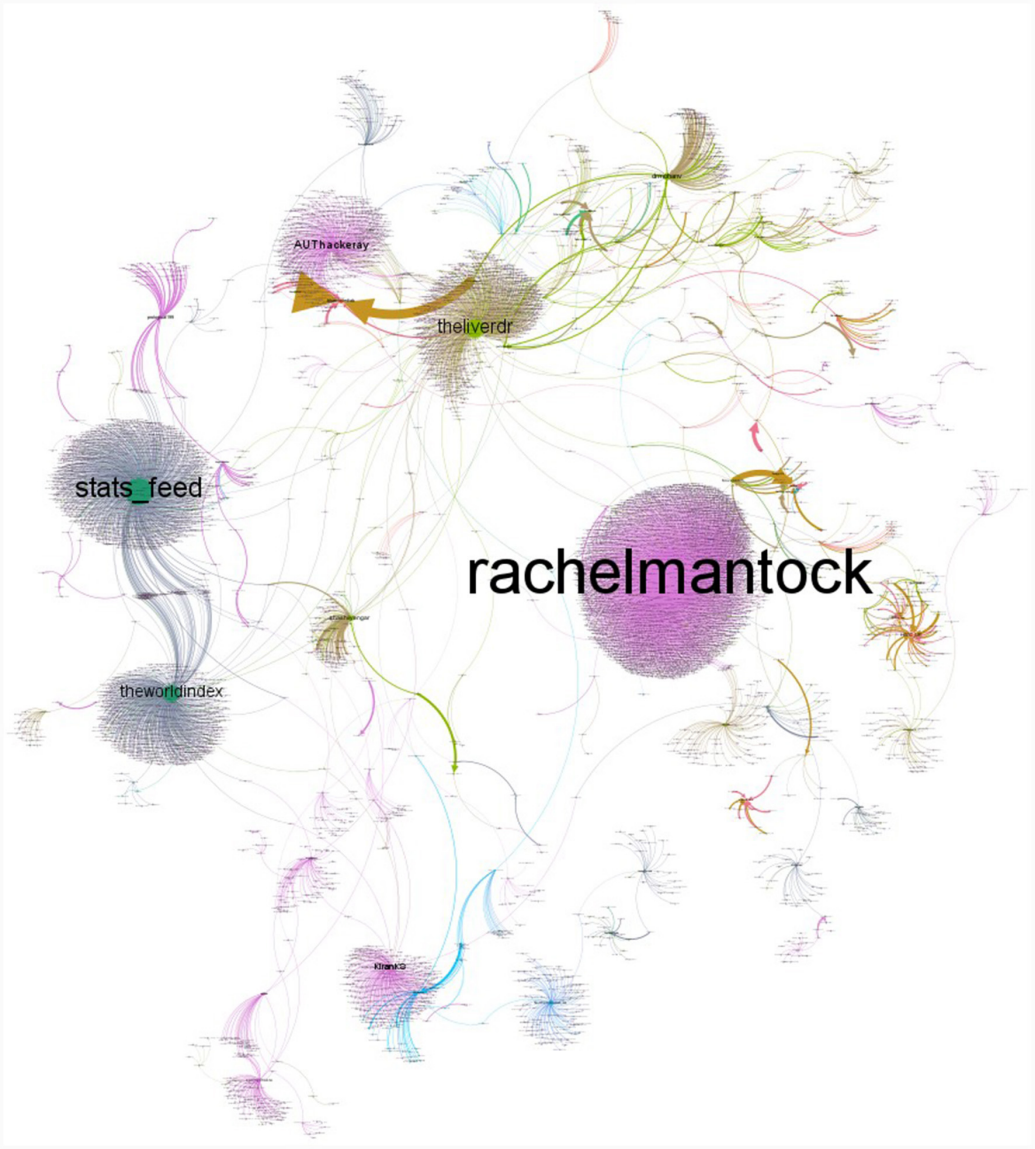
Network of users and influential nodes in diabetes retweet network in India between November 2022 and February 2023.

**Figure 6:**
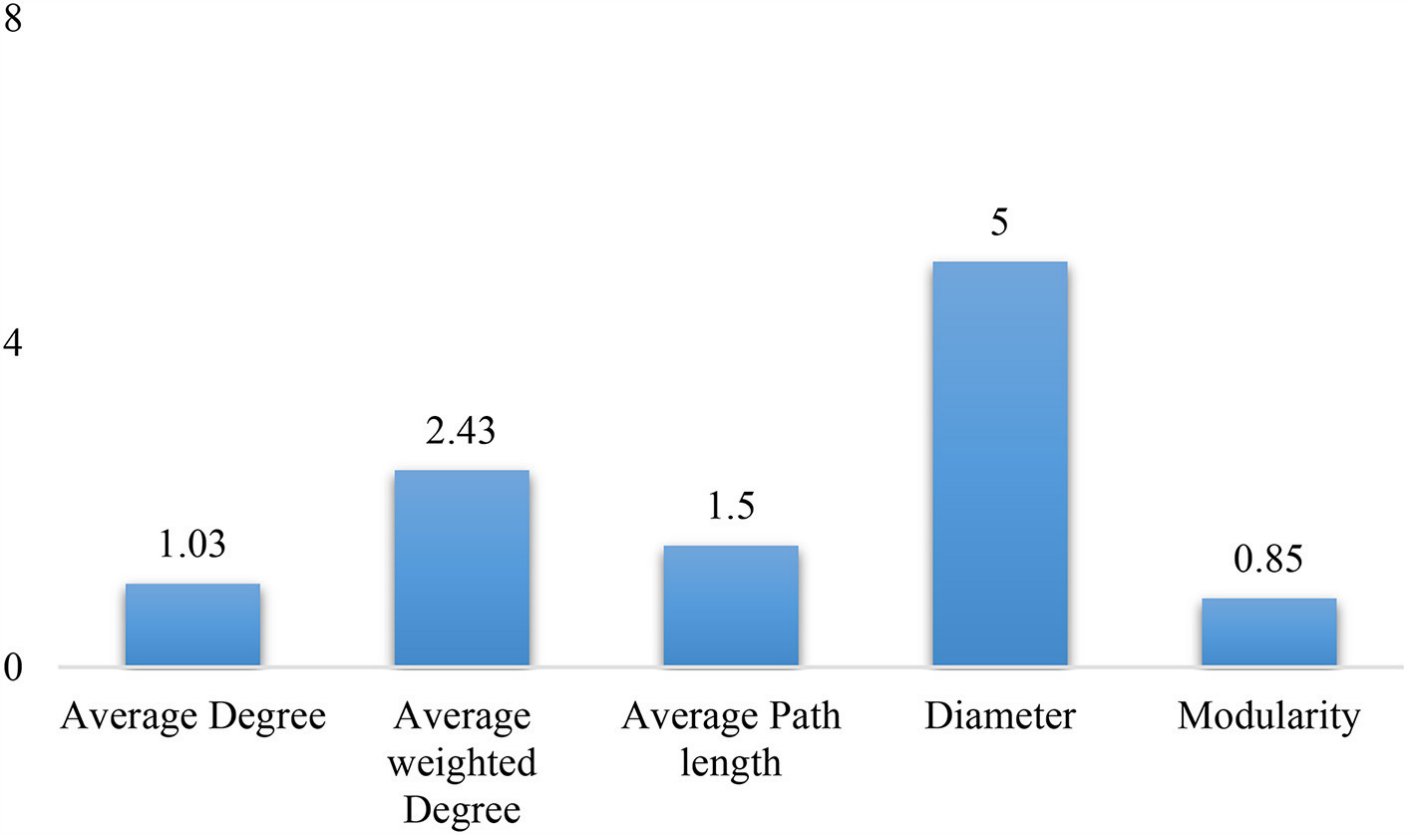
Network statistics for diabetes tweets in India.

There were a total of 18,042 users and 22,067 connections between the users, with degrees ranging from 1 to 2,770 in the retweet along with the mentioned network. Considering the retweet network, there were 7,852 users and 19,364 connections existed. After removing the users with one degree and including only the giant component, there were 6,398 nodes with 15,552 connections considered for further network analysis. Figure 5 shows the overall network of users, with larger circles denoting influential users with a high average weighted degree. On average, the users who tweeted about diabetes interacted with one other users; however, the average weighted degree indicates that there were an average of 2.4 interactions. The average path length of the network is ~1.5 indicating that the information/tweets were passed at least across two users (Figure 6).

The influential users were identified based on metrics such as betweenness centrality, eigenvector centrality, and the PageRank algorithm along with the Hubs and Authorities algorithm. Table 8 lists the top 10 influential users based on various measures. Across the various metrics, individuals belonging to health-related professions (“drmohanv”, “rachelmantock”, “AskDrShashank”,” “dramitaol”, “DrSmitaJoshi2”, “theliverdr”, etc.) majorly constituted the influential group, followed by health organizations (“IYM2023”) and media (“stats_feed”).

**Table 8:** Top 10 influential twitter users in Diabetes network in India.

**Rank**	**Betweeness centrality**		**Eigenvector centrality**		**PageRank algorithm**		**Hubs and Authorities algorithm**	
**Label**	**Value**	**Label**	**Value**	**Label**	**Value**	**Label**	**Value**
1	drmohanv	1,799.0	rachelmantock	1.0000	rachelmantock	0.1493	rachelmantock	1.0000
2	AskDrShashank	948.0	stats_feed	0.4087	stats_feed	0.0583	RORVK	0.0027
3	dramitaol	537.0	theliverdr	0.2915	theliverdr	0.0424	theliverdr	0.0006
4	shashiiyengar	302.0	theworldindex	0.2663	theworldindex	0.0365	LauraMiers	0.0004
5	IYM2023	131.0	AUThackeray	0.1655	AUThackeray	0.0246	RohitChan666	0.0004
6	docanoopmisra	109.0	KiranKS	0.1077	KiranKS	0.0159	vivartist14	0.0004
7	sanjoychakra	67.0	drmohanv	0.0687	alpinelad	0.0091	LifeMathMoney	0.0004
8	anuradhagoyal	59.0	shashiiyengar	0.0612	sanjoychakra	0.0085	AUThackeray	0.0000
9	Pro_Bharati	44.0	sanjoychakra	0.0462	shashiiyengar	0.0063	stats_feed	0.0000
10	banshisaboo	44.0	alpinelad	0.0452	drmohanv	0.0062	theworldindex	0.0000

## 4 Discussion

This study attempted to compare unsupervised machine learning methods of topic modeling to uncover themes in the diabetes-related conversation in India, along with the identification of influential users through network analysis. NMF is a matrix factorization technique that factors the term-document matrix into two lower-dimensional matrices representing topics and term distributions. LDA, on the other hand, factors documents into a mixture of topics. The probabilistic LDA models and the deterministic NMF models are similar in their automatic identification of several topics. In the current study, both models were shown to exhibit almost similar coherence. Although the hyperparameters were chosen carefully, the topics from the LDA model have overlapping topics that are repeated across the identified number of topics (Egger and Yu, 2021). The NMF model is better owing to the TF-IDF weighting and the linear algebraic nature, along with non-negativity constraints that identify latent topics with better accuracy (Egger and Yu, 2022).

The BERTopic and Top2Vec models reduce and cluster the documents based on pre-trained embeddings using the hierarchical structure and identify the topics based on semantic similarity. The topics of the BERTopic and Top2Vec models found that more specific topics were identified in the BERTopic than in the Top2Vec model. There are various embedding models available, and the study leveraged the comparison among them and identified the best-fitting embedding model. The study found that “all-MiniLM-L12-v2” of the BERTopic model had higher coherence.

The study found that the methods NMF and BERTopic do well with analyzing noncommunicable disease-related tweets from India, followed by LDA and Top2Vec. Although BERTopic and NMF provide clear-cut differentiation between the topics identified, the results obtained from NMF were considered to be standard, owing to the coherence of topics identified. The advantage of the BERTopic analysis is its exploration of a keyword-specific topic. Although Top2Vec used pre-trained embedding models, the topics identified overlapped with each other, covering multiple concepts. As a result, this research recommends using BERTopic over NMF to identify topics in short-text Twitter data.

The NMF analysis identified eight latent topics, namely, promotion, management, drug and personal story, consequences, risk factors and research, raising awareness and providing support, diet, opinion, and lifestyle changes. These topics were similar to the manual classification presented by AlBloushi and Abouammoh (2023). The lifestyle change-related tweets have the highest favorite ratio, indicating the likeliness of the readers. However, the lifestyle change-related tweets were less compared to information on risk factors and consequences, which could be increased, which will further improve the awareness of the public. The favorite and retweet ratios were highest for tweets from health-related individuals and lowest for non-health organizations, indicating the reliability and trustworthiness exhibited by the users. This shows that the Twitter platform is highly used for sharing and explaining various diabetes-related information. Similar exploration has been conducted for Indian populations regarding other non-communicable diseases such as Cancer, affirming the effectiveness of social media in this context (Ramamoorthy and Mappillairaju, 2023). Twitter's capacity to enable people to broadcast information and engage with their audiences has made it an excellent platform for sharing information.

The research identified the influential users in spreading diabetes-related information or acting as the main source of information. This indicates that individuals were inclined to share tweets, retweets, and replies from users they considered credible within their professional domains (Kothari et al., 2022). Similar research has been used for the identification of users involved in spreading the conspiracy related to the COVID-19 vaccine, election-based networks, and COVID-19 variants (Ahmed et al., 2020; Chakraborty and Mukherjee, 2023; Yuda Kusuma et al., 2023). Emerging and effective machine learning techniques, including the modified DeepWalk method for link prediction and deep attributed clustering with high-order proximity preservation, leverage both network structure and nodal attributes for prediction and clustering. These approaches delve into aspects less explored in network analysis, offering promising avenues for further research (Berahmand et al., 2021; Berahmand and Li, 2023).

With ever-increasing social media data, the use of topic modeling methods has been increasing but restricted to conventional methods such as LDA, LSA, and NMF (Albalawi et al., 2020). The evolution of topic modeling has given rise to novel methods, and the usage of such methods is recommended to advance information retrieval (Reisenbichler and Reutterer, 2019). This study has taken on the task of comparing four topic modeling methods and suggesting using the least tried embedding-based topic models to encode contextual information, which is not possible through conventional topic models. However, it is important to note that there are many powerful models, such as GPT3 and WuDao, that continue to emerge, and the researchers need to be cognizant of them (Nagisetty, 2021). Twitter has been used as the source of data owing to its 280-character limit. However, any other source of data having similar characteristics shall be analyzed with the current methodology and shall be extendable to any disease of interest.

While our study aimed to delve into conversation dynamics, a limitation arises from the relatively short data collection period of 4 months. This constrained timeframe, while practical for our research objectives, may not capture the full evolution of conversations over an extended period of time. Future research endeavors may benefit from an extended data collection duration to offer a more comprehensive understanding of how conversation dynamics evolve over time. Although the topic models quantified the analysis of short text data, domain knowledge is required for the interpretation of topics. Considering this in mind, this research leveraged the knowledge of the NCD expert in this work. Apart from deriving the content shared related to diabetes, the study identified the influential users, which will be useful for the efficient spread of communication related to diabetes prevention and awareness activities. Regional variations in topics, user engagement, or retweet networks could be studied as a continuation of this research, which can aid in creating targeted interventions.

## Data availability statement

The original contributions presented in the study are included in the article/Supplementary material, further inquiries can be directed to the corresponding author.

## Ethics statement

The studies involving human data were approved by the Ethics Review Committee of the SRM Medical College Hospital and Research Centre - SRM Institute of Science and Technology. Written informed consent was not obtained from the individual(s) for the publication of any potentially identifiable images or data included in this article because publicly accessible posts were analyzed using Twitter's API. The research was conducted in accordance with the local legislation and institutional requirements.

## Author contributions

TR: Conceptualization, Data curation, Formal analysis, Methodology, Software, Validation, Visualization, Writing – original draft, Writing – review & editing. VK: Conceptualization, Data curation, Formal analysis, Methodology, Supervision, Validation, Writing – review & editing. BM: Conceptualization, Supervision, Validation, Writing – review & editing.

## References

[B1] Afroz A. Alramadan M. J. Hossain M. N. Romero L. Alam K. Magliano D.. (2018). Cost-of-illness of type 2 diabetes mellitus in low and lower-middle income countries: a systematic review. BMC Health Serv. Res. 18, 972. 10.1186/s12913-018-3772-8 30558591 PMC6296053

[B2] Ahmed W. Vidal-Alaball J. Downing J. López Segu,í F. (2020). COVID-19 and the 5G conspiracy theory: social network analysis of Twitter data. J. Med. Internet Res. 22:e19458. 10.2196/19458 32352383 PMC7205032

[B3] Alanzi T. (2018). Role of social media in diabetes management in the middle east region: systematic review. J. Med. Internet Res. 20:e58. 10.2196/jmir.9190 29439941 PMC5829453

[B4] Albalawi R. Yeap T. H. Benyoucef M. (2020). Using topic modelling methods for short-text data: a comparative analysis. Front. Artif. Intellig. 3, 42. 10.3389/frai.2020.00042 33733159 PMC7861298

[B5] AlBloushi A. F. Abouammoh M. A. (2023). YouTube videos related to diabetic retinopathy: are they good enough? J. Fr. Ophtalmol. 46, 223–230. 10.1016/j.jfo.2022.07.010 36549928

[B6] Alcoforado A. Ferraz T. P. Gerber R. Bustos E. Oliveira A. S. Veloso B. M.. (2022). “ZeroBERTo - leveraging zero-shot text classification by topic modeling,” in arXiv. (Cham: Fortaleza, Portugal and Springer).

[B7] Angelov D. (2020). Top2Vec: Distributed Representations of Topics. Available online at: http://arxiv.org/pdf/2008.09470v1 (accessed February 12, 2022.).

[B8] Anjana R. M. Unnikrishnan R. Deepa M. Pradeepa R. Tandon N. Das A. K.. (2023). Metabolic non-communicable disease health report of India: the ICMR-INDIAB national cross-sectional study (ICMR-INDIAB-17). Lancet 11, 474–489. 10.1016/S2213-8587(23)00119-5 37301218

[B9] Beguerisse-Díaz M. McLennan A. K. Garduño-Hernández G. Barahona M. (2017). The 'who' and 'what' of #diabetes on Twitter. Digital Health, 3, 2055207616688841. 10.1177/2055207616688841 29942579 PMC6001201

[B10] Berahmand K. Li Y. (2023). and Xu, Y. DAC-HPP: deep attributed clustering with high-order proximity preserve. Neural Comput. Applic. 35, 24493–24511. 10.1007/s00521-023-09052-4

[B11] Berahmand K. Nasiri E. Rostami M. Forouzandeh S. (2021). A modified DeepWalk method for link prediction in attributed social network. Computing 103, 2227–2249. 10.1007/s00607-021-00982-2

[B12] Blei D. M. Ng A. Y. Jordan M. I. (2003). Latent Dirichlet allocation. J. Mach. Learn. Res. 3, 993−1022. Available online at: https://www.jmlr.org/papers/volume3/blei03a/blei03a.pdf

[B13] Cesare N. Oladeji O. Ferryman K. Wijaya D. Hendricks-Muñoz K. D. Ward A.. (2020). Discussions of miscarriage and preterm births on Twitter. Paediatr. Perinatal Epidemiol. 34, 544–552. 10.1111/ppe.12622 31912544 PMC7496231

[B14] Chakraborty A. Mukherjee N. (2023). Analysis and mining of an election-based network using large-scale twitter data: a retrospective study. Soc. Netw. Anal. Min. 13, 74. 10.1007/s13278-023-01081-0 37122615 PMC10115600

[B15] Chen W. Rabhi F. Liao W. Al-Qudah I. (2023). Leveraging state-of-the-art topic modeling for news impact analysis on financial markets: a comparative study. Electronics 12, 2605. 10.3390/electronics12122605

[B16] Chen Y. Zhang H. Liu R. Ye Z. Lin J. (2018). (2019). Experimental explorations on short text topic mining between LDA and NMF based Schemes. Knowl. Based Syst. 163, 1–13. 10.1016/j.knosys.2018.08.011

[B17] Da Moura Semedo C. Bath P. Zhang Z. (2023). Social support in a diabetes online community: mixed methods content analysis. JMIR Diab. 8:e41320. 10.2196/41320 36607714 PMC9945924

[B18] Diviya Prabha V. Rathipriya R. (2022). Diabetes Twitter classification using hybrid GSA. Nature 233, 195. 10.1007/978-3-031-17544-2_9

[B19] Egger R. (2022a). “Text representations and word embeddings. Vectorizing textual data,” in Applied Data Science in Tourism. Interdisciplinary Approaches, Methodologies and Applications, ed. Egger R. (Berlin: Springer), 16.

[B20] Egger R. (2022b). “Topic modelling. Modelling hidden semantic structures in textual data,” in Applied Data Science in Tourism. Interdisciplinary Approaches, Methodologies and Applications, ed. Egger R. (Berlin: Springer), 18.

[B21] Egger R. Yu J. (2021). Identifying hidden semantic structures in Instagram data: a topic modelling comparison. Tour. Rev. 2021, 244. 10.1108/TR-05-2021-0244

[B22] Egger R. Yu J. A. (2022). Topic modeling comparison between LDA, NMF, Top2Vec, and BERTopic to demystify Twitter posts. Front. Sociol. 7:886498. 10.3389/fsoc.2022.886498 35602001 PMC9120935

[B23] Erten M. (2022). HbA1c and e-health: youtube might be good for you, if you use it wisely. Acta. Endocrinol. (Buchar). 18, 531–535. 10.4183/aeb.2022.531 37152888 PMC10162818

[B24] Gabarron E. Dorronzoro E. Rivera-Romero O. (2019). Diabetes on Twitter: a sentiment analysis. J. Diab. Sci. Technol. 13, 439–444. 10.1177/1932296818811679 30453762 PMC6501536

[B25] Gabarron E. Makhlysheva A. (2015). Type 1 Diabetes in Twitter: Who All Listen To?. Stud. Health Technol. Inform. 216, 972. 26262274

[B26] Gavrila V. Garrity A. Hirschfeld E. Edwards B. (2019). Peer support through a diabetes social media community. J. Diabetes sci. Technol. 13, 493–497. 10.1177/1932296818818828 30600704 PMC6501537

[B27] GBD 2019 Diseases and Injuries Collaborators (2020). Global burden of 369 diseases and injuries in 204 countries and territories, 1990–2019: a systematic analysis for the Global Burden of Disease Study 2019. Lancet 396, 1204–1222. 10.1016/S0140-6736(20)30925-9 33069326 PMC7567026

[B28] GBD 2021 Diabetes Collaborators (2023). Global, regional, and national burden of diabetes from 1990 to 2021. with projections of prevalence to 2050: a systematic analysis for the Global Burden of Disease Study 2021. Lancet (London, England) 402, 203–234. 10.1016/S0140-6736(23)01301-6 37356446 PMC10364581

[B29] Ghosh D. D. Guha R. (2013). What are we 'tweeting' about obesity? Mapping tweets with topic modeling and geographic information system. Cartogr. Geographic Information Sci. 40, 90–102. 10.1080/15230406.2013.776210 25126022 PMC4128420

[B30] Greene J. A. Choudhry N. K. Kilabuk E. Shrank W. H. (2011). Online social networking by patients with diabetes: a qualitative evaluation of communication with Facebook. J. Gen. Intern. Med. 26, 287–292. 10.1007/s11606-010-1526-3 20945113 PMC3043192

[B31] Grootendorst M. (2020). BERTopic: Leveraging BERT and c-TF-IDF to Create Easily Interpretable Topics. Zenodo. 10.5281/zenodo.4430182

[B32] Hage P. Harary F. (1995). Eccentricity and centrality in networks. Soc. Netw. 17, 57–63. 10.1016/0378-8733(94)00248-9

[B33] Haghravan S. Mohammadi-Nasrabadi F. Rafraf M. A. (2021). critical review of national diabetes prevention and control programs in 12 countries in Middle East. Diabetes Metab. Syndr. Clin. Res. Rev. 15, 439–45. 10.1016/j.dsx.2021.02.002 33592370

[B34] Hendry D. Darari F. Nurfadillah R. Khanna G. Sun M. Condylis P. C.. (2021). “Topic modeling for customer service chats,” in 2021 International Conference on Advanced Computer Science and Information Systems (Piscataway, NJ: IEEE), 1–6.

[B35] Karami A. Dahl A. A. Turner-McGrievy G. Kharrazi H. Shaw G. (2018). Characterizing diabetes, diet, exercise, and obesity comments on Twitter. Int. J. Inform. Manage. 38, 1–6. 10.1016/j.ijinfomgt.2017.08.002

[B36] Karmegam D. (2022). Social media analytics and reachability evaluation - #Diabetes. Diab. Metab. Synd. 16, 102359. 10.1016/j.dsx.2021.102359 34920205

[B37] Kothari A. Walker K. Burns K. (2022). # CoronaVirus and public health: the role of social media in sharing health information. OIR 46, 1293–1312. 10.1108/OIR-03-2021-0143

[B38] Kulothungan V. Ramamoorthy T. Mohan R. Mathur P. (2023). Assessing progress of India in reduction of premature mortality due to four noncommunicable diseases towards achieving the WHO 25_25 goal and the sustainable development goals. Sustain. Dev. 1–11. 10.1002/sd.2761

[B39] Lenzi F. R. Iazzetta F. (2023). Mapping obesity and diabetes' representation on Twitter: the case of Italy. Front. Sociol. 8, 1155849. 10.3389/fsoc.2023.1155849 37397627 PMC10311219

[B40] Liu Y. Mei Q. Hanauer D. Zheng K. Lee J. (2016). Use of social media in the diabetes community: an exploratory analysis of diabetes-related tweets. JMIR Diab. 1, e4. 10.2196/diabetes.6256 30291053 PMC6238851

[B41] Ma P. Zeng-Treitler Q. Nelson S. J. (2021). Use of two topic modelling methods to investigate covid vaccine hesitancy. Int. Conf. ICT Soc. Hum. Beings 384, 221–226. Available online at: https://www.ict-conf.org/wp-content/uploads/2021/07/04_202106C030_Ma.pdf

[B42] Moorhead S. A. Hazlett D. E. Harrison L. Carroll J. K. Irwin A. Hoving C. A.. (2013). new dimension of health care: systematic review of the uses, benefits, and limitations of social media for health communication. J. Med. Internet Res. 15:e85. 10.2196/jmir.1933 23615206 PMC3636326

[B43] Murshed B. A. H. Mallappa S. Abawajy J. Saif M. A. N. Al-Ariki H. D. E. Abdulwahab H. M.. (2023). Short text topic modelling approaches in the context of big data: taxonomy, survey, and analysis. Artif. Intell. Rev. 56, 5133–5260. 10.1007/s10462-022-10254-w 36320612 PMC9607740

[B44] Nagisetty V. (2021). Domain Knowledge Guided Testing and Training of Neural Networks. (Master's thesis) (Waterloo, ON: University of Waterloo).

[B45] Obadimu A. Mead E. Agarwal N. (2019). “Identifying latent toxic features on YouTube using non-negative matrix factorization,” in The Ninth International Conference on Social Media Technologies, Communication, and Informatics (Valencia: Ninth International Conference), 1–6.

[B46] Park H. Reber B. H. Chon M.-G. (2015). Tweeting as health communication: health organizations' use of Twitter for health promotion and public engagement. J. Health Commun. 21, 188–198. 10.1080/10810730.2015.1058435 26716546

[B47] Petrosyan A. (2023). Internet and Social Media Users in the World 2023. Statista. Available online at: https://www.statista.com/statistics/617136/digital-population-worldwide/ (accessed May 25, 2023).

[B48] Probabilistic Topic Models (2021). Communications of the ACM. Available online at: https://dl.acm.org/doi/fullHtml/10, 1145./2133806.2133826 (accessed 27 September, 2021).

[B49] Raamkumar A. S. Pang N. Foo S. (2016). When countries become the talking point in microblogs: study on country hashtags in Twitter | First Monday. Clin. Hemorheol. Microcirc. 21, 1–4. 10.5210/fm.v21i1.6101

[B50] Ramamoorthy T. Mappillairaju B. (2023). Tweet topics on cancer among Indian Twitter users-computational approach using latent Dirichlet allocation topic modelling. J. Comput. Soc. Sci. 6, 1033–1054. 10.1007/s42001-023-00222-x

[B51] Rana A. Arora M. (2023) Ketogenic diet: assessing YouTube video information using quality, reliability, text analytics methods. Nutr. Health. 2023, 2601060231193789. 10.1177/02601060231193789 37559420

[B52] Reisenbichler M. Reutterer T. (2019). Topic modeling in marketing: recent advances and research opportunities. J. Bus. Econ. 89, 327–356. 10.1007/s11573-018-0915-7

[B53] Shaw G. Karami A. (2017). Computational content analysis of negative tweets for obesity, diet, diabetes, and exercise. Proc. Assoc. Inf. Sci. Technol. 54, 357e.65. 10.1002/pra2.2017.14505401039

[B54] Siegel K. R. Patel S. A. Ali M. K. (2014). Non-communicable diseases in South Asia: contemporary perspectives. Br. Med. Bull. 111, 31–44. 10.1093/bmb/ldu018 25190759 PMC4416117

[B55] Smailhodzic E. Hooijsma W. Boonstra A.. (2016). Social media use in healthcare: A systematic review of effects on patients and on their relationship with healthcare professionals. BMC Health Serv. Res. 16:442. 10.1186/s12913-016-1691-0 27562728 PMC5000484

[B56] Staite E. Zaremba N. Macdonald P. Allan J. Treasure J. Ismail K.. (2018). ‘Diabulima' through the lens of social media: a qualitative review and analysis of online blogs by people with Type 1 diabetes mellitus and eating disorders. Diabet. Med. 35:1329. 10.1111/dme.13700 29855073

[B57] Stellefson M. Paige S. Apperson A. Spratt S. (2019). Social media content analysis of public diabetes Facebook groups. J. Diabetes Sci. Technol. 13, 428–438. 10.1177/1932296819839099 30931593 PMC6501525

[B58] Stevens K. Kegelmeyer P. Andrzejewski D. Buttler D. (2012). “Exploring topic coherence over many models and many topics,” in Proceedings of the 2012 Joint Conference on Empirical Methods in Natural Language Processing and Computational Natural Language Learning (Jeju Island, Korea: Joint Conference on Empirical Methods), 952–961.

[B59] Tapi Nzali M. D. Bringay S. Lavergne C. Mollevi C. (2017). What patients can tell us: topic analysis for social media on breast cancer. JMIR Med. Inform. 5:e23. 10.2196/medinform.7779 28760725 PMC5556259

[B60] The Healthcare Hashtag Project (2023). Symplur. Available online at: https://www.symplur.com/healthcare-hashtags/ (accessed 23 April, 2023).

[B61] The World Bank (2013). “The global burden of disease: generating evidence, guiding policy—south Asia regional edition,” in Institute for Health Metrics and Evaluation, Human Development Network, The World Bank. Seattle, WA: IHME.

[B62] Thielmann A. F. Weisser C. Kneib T. Saefken B. (2021). “Coherence based document clustering,” in The International Conference on Learning Representations, 1–14.

[B63] Tripathy J. P. Sagili K. D. Kathirvel S. Trivedi A. Nagaraja S. B. Bera O. P.. (2019). Diabetes care in public health facilities in India: a situational analysis using a mixed methods approach. Diabetes Metab. Syndr. Obes. 12, 1189–1199. 10.2147/DMSO.S192336 31410044 PMC6650449

[B64] Twitter Developer (2023). About Twitter API. Availoable online at: https://developer.twitter.com/en/docs/twitter-api/getting-started/ about-twitter-api (accessed March 1, 2023).

[B65] Valdez D. Ten Thij M. Bathina K. Rutter L. A. (2020). Social media insights into US mental health during the COVID-19 pandemic: longitudinal analysis of Twitter data. J. Med. Internet Res. 22:e21418. 10.2196/21418 33284783 PMC7744146

[B66] White R. O. Eden S. Wallston K. A. Kripalani S. Barto S. Shintani A.. (2014). (2015). Health communication, self-care, and treatment satisfaction among low-income diabetes patients in a public health setting. Patient Educ. Counsel. 98:144e9. 10.1016/j.pec.2014.10.019 25468393 PMC4282939

[B67] Yu J. Egger R. (2021). Color and engagement in touristic Instagram pictures: a machine learning approach. Ann. Tour. Res. 2021:103204. 10.1016/j.annals.2021.103204

[B68] Yuda Kusuma I. Pratiwi H. Fitri Khairunnisa S. Ayu Eka Pitaloka D. Arizandi Kurnianto A. (2023). The assessment of Twitter discourse on the new COVID-19 variant, XBB.1.5, through social network analysis. Vaccine X 14, 100322. 10.1016/j.jvacx.2023.100322 37317688 PMC10245456

[B69] Zhou S. Zhao Y. Bian J. Haynos A. F. Zhang R. (2020). Exploring eating disorder topics on twitter: machine learning approach. JMIR Med. Inform. 8:e18273. 10.2196/18273 33124997 PMC7665945

