# Supplementary material for: Topic modeling and social network analysis approach to explore diabetes discourse on Twitter in India
Source: Front Artif Intell. 2024 Feb 12;7:1329185. doi: 10.3389/frai.2024.1329185 (PMC10895681; doi:10.3389/frai.2024.1329185)
Supplement: Supplementary file 1 [file Data_Sheet_1.docx]

**Appendix Figure 1: Coherence scores for the different number of topics for Diabetes related tweets in India**

**Appendix Figure 2: Intertopic distance map for diabetes related tweets in India**

**
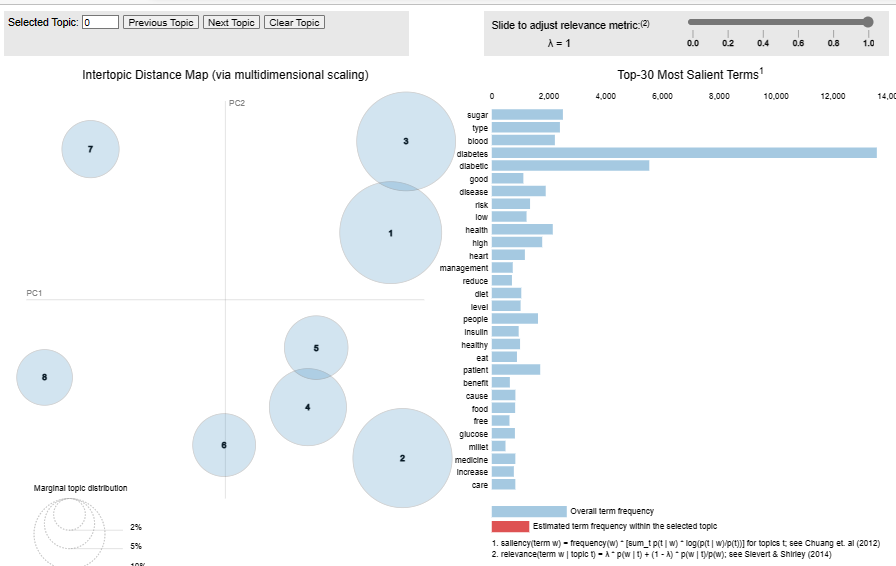
**

**Appendix Figure 3: t-distributed stochastic neighbor embedding clustering Visualized for diabetes related tweets in India**

**
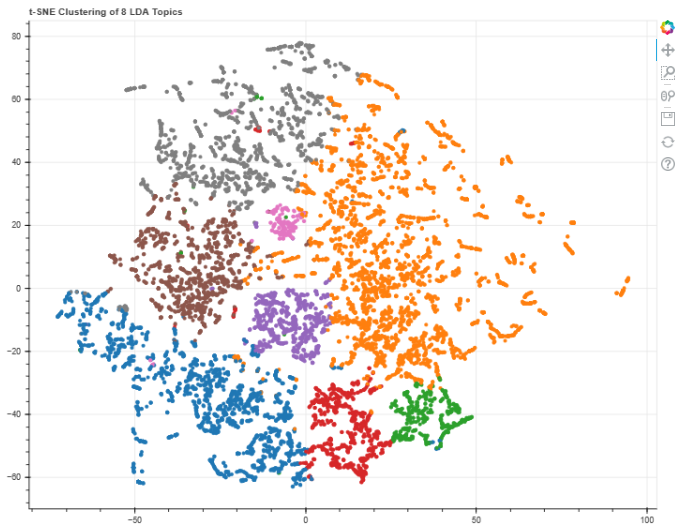
**

**Appendix Figure 4: Frequency of diabetes related tweets in India during the study period (November 2022 – February 2023)**

**Appendix Figure 5**: Distribution (%) of type of user tweeted regarding diabetes in India during the study period **(November 2022 – February 2023)**

Appendix Figure 6: Intertopic distance map created using BERTopic method for the diabetes tweets in India


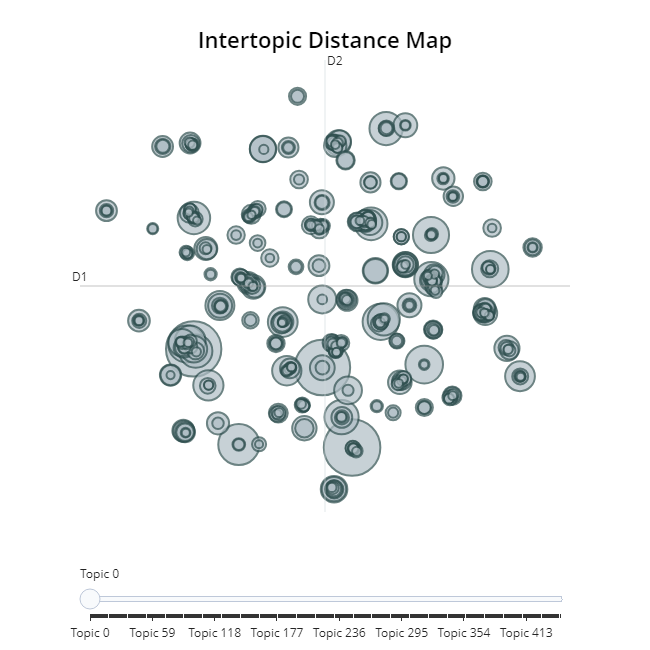


**Appendix table 1: List of top 50 probability (beta) distributions of words in each diabetes related topic identified.**

| No of words | Topic 1 | | | | |  | Topic 2 | | | | |  | Topic 3 | | | | |  | Topic 4 | | |  |
| --- | --- | --- | --- | --- | --- | --- | --- | --- | --- | --- | --- | --- | --- | --- | --- | --- | --- | --- | --- | --- | --- | --- |
|  | Word | | Beta | | |  | Word | | Beta | | |  | Word | | Beta | | |  | Word | Beta | |  |
| diabetes | | 0.047838148 | | |  | year | | 0.0358805 | | |  | diabetic | | 0.1086427 | | |  | diabetes | 0.083357 | |  |  |
| 2 | day | | 0.028183846 | | |  | age | | 0.0276773 | | |  | diabetes | | 0.0356428 | | |  | health | 0.0406075 | |  |
| 3 | world | | 0.02359569 | | |  | drug | | 0.0218878 | | |  | patient | | 0.0306436 | | |  | disease | 0.03957 | |  |
| 4 | start | | 0.01901324 | | |  | patient | | 0.0214521 | | |  | good | | 0.027652 | | |  | risk | 0.025088 | |  |
| 5 | research | | 0.018751342 | | |  | change | | 0.0172013 | | |  | level | | 0.024224 | | |  | india | 0.0230129 | |  |
| 6 | stress | | 0.016826449 | | |  | drink | | 0.0166299 | | |  | control | | 0.0184366 | | |  | type | 0.0219092 | |  |
| 7 | watch | | 0.016127896 | | |  | family | | 0.0154614 | | |  | management | | 0.017244 | | |  | heart | 0.0211167 | |  |
| 8 | reverse | | 0.015509245 | | |  | post | | 0.015009 | | |  | foot | | 0.0168522 | | |  | medicine | 0.0183798 | |  |
| 9 | sir | | 0.014588573 | | |  | citizen | | 0.0133795 | | |  | obesity | | 0.0167032 | | |  | weight | 0.0157265 | |  |
| 10 | book | | 0.014419529 | | |  | vs | | 0.0129644 | | |  | life | | 0.01542 | | |  | increase | 0.014711 | |  |
| 11 | th | | 0.010775931 | | |  | share | | 0.0122698 | | |  | problem | | 0.0150594 | | |  | study | 0.0137677 | |  |
| 12 | join | | 0.010660323 | | |  | claim | | 0.0117125 | | |  | treatment | | 0.0146729 | | |  | non | 0.0123306 | |  |
| 13 | meal | | 0.010334881 | | |  | tell | | 0.0106544 | | |  | help | | 0.0136205 | | |  | benefit | 0.0113708 | |  |
| 14 | result | | 0.009958684 | | |  | till | | 0.0103875 | | |  | check | | 0.0127104 | | |  | lead | 0.0111176 | |  |
| 15 | Right | | 0.009855419 | | |  | system | | 0.0093726 | | |  | use | | 0.0105436 | | |  | condition | 0.009055 | |  |
| 16 | lack | | 0.009761515 | | |  | product | | 0.0093286 | | |  | treat | | 0.0103897 | | |  | hypertension | 0.0090235 | |  |
| 17 | solution | | 0.00917847 | | |  | adult | | 0.0092646 | | |  | find | | 0.0102397 | | |  | link | 0.0089374 | |  |
| 18 | physician | | 0.009066046 | | |  | young | | 0.008805 | | |  | improve | | 0.0090359 | | |  | bp | 0.0089113 | |  |
| 19 | appointment | | 0.008975758 | | |  | normal | | 0.0082677 | | |  | eye | | 0.0086691 | | |  | high | 0.0087849 | |  |
| 20 | relate | | 0.008318624 | | |  | impact | | 0.007768 | | |  | think | | 0.0086658 | | |  | body | 0.0086802 | |  |
| 21 | fight | | 0.00812427 | | |  | general | | 0.0072566 | | |  | early | | 0.0083329 | | |  | people | 0.007977 | |  |
| 22 | clinic | | 0.008035759 | | |  | consider | | 0.0071368 | | |  | market | | 0.0072736 | | |  | medical | 0.0076963 | |  |
| 23 | science | | 0.007529307 | | |  | vegetable | | 0.0069234 | | |  | follow | | 0.0072733 | | |  | factor | 0.0076948 | |  |
| 24 | airport | | 0.00734521 | | |  | surgery | | 0.0068506 | | |  | add | | 0.007233 | | |  | loss | 0.0072902 | |  |
| 25 | online | | 0.007281879 | | |  | device | | 0.0068135 | | |  | tip | | 0.007228 | | |  | chronic | 0.0068331 | |  |
| 26 | bring | | 0.007211869 | | |  | rural | | 0.0068024 | | |  | experience | | 0.0071129 | | |  | suffer | 0.0064105 | |  |
| 27 | speak | | 0.007117424 | | |  | milk | | 0.0062689 | | |  | thing | | 0.0069111 | | |  | develop | 0.0061896 | |  |
| 28 | hour | | 0.006905362 | | |  | hyperglycemia | | 0.0062488 | | |  | tea | | 0.006613 | | |  | case | 0.0061397 | |  |
| 29 | present | | 0.006895269 | | |  | screen | | 0.0061287 | | |  | expert | | 0.0066062 | | |  | issue | 0.0060932 | |  |
| 30 | community | | 0.006895103 | | |  | area | | 0.006114 | | |  | live | | 0.0065154 | | |  | cancer | 0.0058521 | |  |
| 31 | second | | 0.006603019 | | |  | information | | 0.0061042 | | |  | bad | | 0.0063777 | | |  | kidney | 0.0057008 | |  |
| 32 | power | | 0.006266432 | | |  | pay | | 0.0060548 | | |  | long | | 0.0062809 | | |  | mellitus | 0.0055593 | |  |
| 33 | let | | 0.006142762 | | |  | hon | | 0.0058082 | | |  | come | | 0.0059953 | | |  | person | 0.005438 | |  |
| 34 | conclave | | 0.006120751 | | |  | clinical | | 0.0053835 | | |  | lose | | 0.0055053 | | |  | metabolic | 0.0052852 | |  |
| 35 | video | | 0.005842648 | | |  | safe | | 0.005313 | | |  | symptom | | 0.0054435 | | |  | available | 0.0051312 | |  |
| 36 | launch | | 0.005696833 | | |  | game | | 0.0052572 | | |  | major | | 0.0054149 | | |  | water | 0.004972 | |  |
| 37 | camp | | 0.005472682 | | |  | mental | | 0.0050177 | | |  | understand | | 0.0053764 | | |  | cure | 0.0048268 | |  |
| 38 | association | | 0.005317073 | | |  | half | | 0.004955 | | |  | prevent | | 0.0053318 | | |  | sleep | 0.0048176 | |  |
| 39 | chapter | | 0.005302377 | | |  | respect | | 0.0047718 | | |  | complication | | 0.0052959 | | |  | capital | 0.0046907 | |  |
| 40 | history | | 0.005007869 | | |  | yrs | | 0.0047041 | | |  | happen | | 0.0050307 | | |  | work | 0.004679 | |  |
| 41 | wellness | | 0.00499324 | | |  | surprise | | 0.0046077 | | |  | look | | 0.0049501 | | |  | common | 0.0046537 | |  |
| 42 | protect | | 0.004960837 | | |  | ble | | 0.0043919 | | |  | retinopathy | | 0.0044944 | | |  | bone | 0.0046228 | |  |
| 43 | special | | 0.004875862 | | |  | village | | 0.004374 | | |  | poor | | 0.0043461 | | |  | effect | 0.0046176 | |  |
| 44 | mumbai | | 0.004842317 | | |  | metformin | | 0.0042196 | | |  | healthcare | | 0.0042197 | | |  | associate | 0.0045319 | |  |
| 45 | session | | 0.0047875 | | |  | researcher | | 0.0041158 | | |  | habit | | 0.0041949 | | |  | cardiovascular | 0.004424 | |  |
| 46 | buy | | 0.00476599 | | |  | urban | | 0.0040817 | | |  | range | | 0.0041227 | | |  | know | 0.0043259 | |  |
| 47 | create | | 0.004761167 | | |  | incidence | | 0.0039963 | | |  | spike | | 0.0040466 | | |  | grow | 0.003821 | |  |
| 48 | discussion | | 0.00475552 | | |  | ignore | | 0.0039774 | | |  | point | | 0.0039896 | | |  | monitoring | 0.0038195 | |  |
| 49 | soon | | 0.00463124 | | |  | male | | 0.0039419 | | |  | sensitivity | | 0.0039768 | | |  | attack | 0.0037673 | |  |
| 50 | pm | | 0.004565106 | | |  | rd | | 0.0038651 | | |  | ayurvedic | | 0.0039522 | | |  | app | 0.0037396 | |  |
| No of words | Topic 5 | | |  | Topic 6 | | | | |  | Topic 7 | | | | |  | Topic 8 | | | | | |
|  | Word | Beta | |  | Word | | | Beta | |  | Word | | | Beta | |  | Word | | | |  |  |
| 1 | diabetes | 0.1236797 | |  | sugar | | | 0.0650137 | |  | rice | | | 0.0683657 | |  | reduce | | | | 0.0340259 | |
| 2 | type | 0.0301057 | |  | blood | | | 0.0561916 | |  | oil | | | 0.041985 | |  | time | | | | 0.0220959 | |
| 3 | healthy | 0.0257907 | |  | diabetic | | | 0.0400587 | |  | pre | | | 0.0296221 | |  | protein | | | | 0.020165 | |
| 4 | doctor | 0.0235462 | |  | diabetes | | | 0.0367585 | |  | technology | | | 0.0283166 | |  | stop | | | | 0.0184931 | |
| 5 | need | 0.0226906 | |  | high | | | 0.0342664 | |  | glycemic | | | 0.0197051 | |  | fat | | | | 0.0168257 | |
| 6 | cause | 0.0219241 | |  | low | | | 0.0316795 | |  | south | | | 0.0177229 | |  | covid | | | | 0.0131984 | |
| 7 | care | 0.021299 | |  | diet | | | 0.0236646 | |  | option | | | 0.0156425 | |  | try | | | | 0.0121244 | |
| 8 | people | 0.0175208 | |  | eat | | | 0.0228916 | |  | wheat | | | 0.0140734 | |  | number | | | | 0.0119132 | |
| 9 | lifestyle | 0.0167429 | |  | glucose | | | 0.022333 | |  | breakfast | | | 0.0131524 | |  | regular | | | | 0.011797 | |
| 10 | manage | 0.014463 | |  | food | | | 0.0209317 | |  | education | | | 0.0097751 | |  | rich | | | | 0.0114036 | |
| 11 | hospital | 0.0115177 | |  | insulin | | | 0.0201885 | |  | course | | | 0.0096511 | |  | hypoglycemia | | | | 0.010702 | |
| 12 | sweet | 0.0111416 | |  | people | | | 0.0133769 | |  | balance | | | 0.0096401 | |  | sign | | | | 0.0103878 | |
| 13 | way | 0.0108995 | |  | cholesterol | | | 0.0115939 | |  | taste | | | 0.0096309 | |  | pain | | | | 0.0100411 | |
| 14 | read | 0.0089296 | |  | millet | | | 0.0113098 | |  | wedding | | | 0.0095264 | |  | natural | | | | 0.0097636 | |
| 15 | population | 0.0083347 | |  | help | | | 0.0110927 | |  | govt | | | 0.0092988 | |  | pharma | | | | 0.0096138 | |
| 16 | ask | 0.0078084 | |  | free | | | 0.0110919 | |  | quick | | | 0.0087362 | |  | friendly | | | | 0.0095543 | |
| 17 | important | 0.0075935 | |  | vegetarian | | | 0.0100126 | |  | dear | | | 0.0083593 | |  | promote | | | | 0.0095425 | |
| 18 | senior | 0.0067602 | |  | pressure | | | 0.0089489 | |  | choose | | | 0.0082162 | |  | rate | | | | 0.0092535 | |
| 19 | level | 0.0066091 | |  | state | | | 0.0088765 | |  | wake | | | 0.0079485 | |  | tablet | | | | 0.008586 | |
| 20 | woman | 0.0065219 | |  | include | | | 0.0084081 | |  | roti | | | 0.0079197 | |  | morning | | | | 0.0083224 | |
| 21 | gestational | 0.0064999 | |  | test | | | 0.0081261 | |  | fatty | | | 0.0077265 | |  | patient | | | | 0.0082775 | |
| 22 | million | 0.0063618 | |  | carb | | | 0.0077031 | |  | contribute | | | 0.0070854 | |  | alcohol | | | | 0.0082142 | |
| 23 | month | 0.0062642 | |  | leave | | | 0.0076435 | |  | thank | | | 0.0068026 | |  | fit | | | | 0.0077002 | |
| 24 | support | 0.0062525 | |  | company | | | 0.0076134 | |  | large | | | 0.0067082 | |  | away | | | | 0.0076834 | |
| 25 | control | 0.0061389 | |  | consume | | | 0.0073115 | |  | ailment | | | 0.00654 | |  | wait | | | | 0.007296 | |
| 26 | stay | 0.0061285 | |  | great | | | 0.0072371 | |  | ad | | | 0.006481 | |  | physical | | | | 0.0068387 | |
| 27 | prediabetes | 0.0059476 | |  | apple | | | 0.0072142 | |  | season | | | 0.0064029 | |  | contain | | | | 0.0064598 | |
| 28 | country | 0.0058918 | |  | visit | | | 0.0070443 | |  | suitable | | | 0.0061908 | |  | daily | | | | 0.006213 | |
| 29 | enjoy | 0.0057748 | |  | consumption | | | 0.0070369 | |  | dementia | | | 0.0055767 | |  | compare | | | | 0.0061807 | |
| 30 | learn | 0.0057271 | |  | vitamin | | | 0.007019 | |  | minute | | | 0.0054603 | |  | sell | | | | 0.0061644 | |
| 31 | liver | 0.005714 | |  | exercise | | | 0.0069686 | |  | worldwide | | | 0.0054353 | |  | walk | | | | 0.0061598 | |
| 32 | lot | 0.0055545 | |  | medication | | | 0.0060994 | |  | nation | | | 0.0053469 | |  | role | | | | 0.0061204 | |
| 33 | prevalence | 0.0053334 | |  | fruit | | | 0.0060035 | |  | job | | | 0.0052519 | |  | powder | | | | 0.0057665 | |
| 34 | home | 0.0052656 | |  | meat | | | 0.0059687 | |  | middle | | | 0.0052406 | |  | fibre | | | | 0.0057418 | |
| 35 | child | 0.0051877 | |  | provide | | | 0.0052369 | |  | nutritional | | | 0.0051504 | |  | almond | | | | 0.0057269 | |
| 36 | talk | 0.0051131 | |  | disorder | | | 0.0051511 | |  | acid | | | 0.0051031 | |  | prescribe | | | | 0.0056441 | |
| 37 | coffee | 0.0051008 | |  | mean | | | 0.0051048 | |  | face | | | 0.0050288 | |  | onset | | | | 0.0054635 | |
| 38 | diagnose | 0.0050464 | |  | cost | | | 0.0050406 | |  | resistant | | | 0.0050084 | |  | beneficial | | | | 0.0052764 | |
| 39 | easy | 0.0049418 | |  | map | | | 0.0049587 | |  | se | | | 0.0047769 | |  | kg | | | | 0.005225 | |
| 40 | nutrition | 0.0047692 | |  | fast | | | 0.0048977 | |  | bean | | | 0.0047618 | |  | suffering | | | | 0.0051671 | |
| 41 | yes | 0.0046645 | |  | hba | | | 0.0048153 | |  | prefer | | | 0.0046955 | |  | multi | | | | 0.0050929 | |
| 42 | today | 0.0044436 | |  | monitor | | | 0.0047957 | |  | dr | | | 0.0046287 | |  | similar | | | | 0.0049131 | |
| 43 | help | 0.0042488 | |  | small | | | 0.0044311 | |  | content | | | 0.004545 | |  | interesting | | | | 0.0046914 | |
| 44 | happy | 0.0041462 | |  | grain | | | 0.0042824 | |  | tamil | | | 0.0045201 | |  | price | | | | 0.0045658 | |
| 45 | team | 0.0041248 | |  | plan | | | 0.0041804 | |  | dish | | | 0.0044011 | |  | aid | | | | 0.0045175 | |
| 46 | north | 0.0040188 | |  | veg | | | 0.004072 | |  | ready | | | 0.004364 | |  | memory | | | | 0.0044219 | |
| 47 | place | 0.0040083 | |  | excess | | | 0.0040346 | |  | thousand | | | 0.0041992 | |  | death | | | | 0.0043777 | |
| 48 | feel | 0.0037938 | |  | index | | | 0.0040326 | |  | bharat | | | 0.0040659 | |  | guy | | | | 0.0043715 | |
| 49 | superfood | 0.0037901 | |  | regulate | | | 0.0038757 | |  | green | | | 0.0040484 | |  | vaccine | | | | 0.0043412 | |
| 50 | friend | 0.0037562 | |  | manage | | | 0.0038282 | |  | gt | | | 0.0039859 | |  | vegan | | | | 0.0042895 | |
|  |  |  | |  |  | | |  | |  |  | | |  | |  |  | | | |  | |
